# Editorial: Novel approaches aiming to overcome current nanomedicine limitations

**DOI:** 10.3389/fmolb.2023.1247959

**Published:** 2023-07-05

**Authors:** Roberto Palomba, Michele Schlich, Michael Evangelopoulos, Raffaele Spanò

**Affiliations:** ^1^ Laboratory of Nanotechnology for Precision Medicine, Italian Institute of Technology, Genoa, Italy; ^2^ Dipartimento di Scienze della Vita e dell'Ambiente, Università di Cagliari, Cagliari, Italy; ^3^ Regenerative Medicine Program, Houston Methodist Research Institute, Houston, TX, United States

**Keywords:** nanomedicine, GBM-glioblastoma multiforme, sarcoma, ocular delivery, atherosclerosis

The Research Topic *Novel approaches aiming to overcome current nanomedicine limitations* was conceived with the idea of collecting original articles and reviews highlighting innovative strategies being used to increase the effectiveness of nanoformulations in therapy and diagnosis. Herein, we provide a brief synopsis of the articles included in this Research Topic: two reviews presenting novel nanomedicine-based approaches for the treatment of glioblastoma and sarcoma, one review highlighting the advancements made in nanomedicines for ocular-specific applications, and an original article describing a novel nanosystem targeting atherosclerotic plaques.

Allami et al. contribute to this Research Topic with a review encompassing the possibilities offered by functionalizing nanoparticle surfaces with cellular-derived membranes; the review focuses on the application of these strategies for the treatment of glioblastoma (GBM). After presenting the main pathological characteristics of GBM, together with the current methods for diagnosis and treatment, the authors then describe challenges in delivering drugs across the blood brain barrier (BBB)—to date, a major hurdle for the successful treatment of this pathology. The authors present an outline of nanotechnology as applied to cancer with an overview of various nanocarrier platforms. The review continues by highlighting recent preclinical studies of nanocarriers, which enable the development of novel methods that could facilitate the delivery of therapeutics across the BBB. In this regard, functionalizing the surface of nanoparticles with cellular membrane provides a promising approach. The common steps required for this process are discussed and a series of possible sources of coating is also described: from erythrocytes to dendritic cells, moving through neutrophils, macrophages, and other immune cells, and finally cancer cells. Other sources were also discussed, such as platelets, bacteria, and specific membrane organelles. A section describing the use of these methods for the treatment of GBM follows with a subsection dedicated to chemotherapy, immunotherapy, gene therapy and synergistic phototherapy.

Mercatali et al. add to this Research Topic with a review presenting the application of nanomedicine to target connective tissue cancers. After describing the biology of sarcomas, the authors introduce the current therapeutic strategies for its treatment. The nanotechnology-based platforms available in translational research are then discussed. Both active and passive targeting formulations are highlighted with a subsection dedicated to novel formulations being investigated for the treatment of osteosarcoma. The discussion then focuses on: i) the application of lab-on-a-chip devices as promising platforms for diagnosis, prognosis, and screening of sarcomas and ii) novel 3D culture systems dedicated to the study of sarcoma physiopathology. The authors next highlight the small but significant steps carried out in translational research over the past decade by discussing clinical trials that explored the use of nanomedicines applied to sarcoma. Particular attention was dedicated to the first-in-class radio-enhancer, hafnium oxide nanoparticle (NBTXR3), which was recently introduced in a phase 2–3 clinical trial. Finally, ongoing clinical trials assessing the efficacy of other drug delivery systems in sarcoma are then described.

A contribution to this Research Topic by Yang et al. reviews the use of nanoparticles for ocular-related applications. The focus of this work was on the risk of toxicity that might be induced by nanoparticle delivery to the eye. After a brief introduction, authors analyze the different types of nanoparticles specifically developed for the treatment of anterior and posterior segment diseases. Nanoparticles are classified based on their composition, with subdivisions into well-known categories. Each class was briefly defined, highlighting the rationale for their use against eye diseases. Following a paragraph on the application of nanoparticles for diagnostic purposes in ophthalmology, the authors delve into ocular-focused applications of nanoparticles, classifying the studies in four pathological areas: oxidative stress, inflammation, angiogenesis, and infection. Lastly, another section was dedicated to safety. Here, the studies were again classified based on the nanoparticle type, since the composition and material quality are intimately linked to specific risks (i.e., accumulation of heavy metals in the cornea following the administration of quantum dots) or to a satisfactory tolerability. In the last section, the authors provide their expert opinion on what they consider to be the main limitation of the field; that is, the insufficient investigation of the safety of nanocarriers for ocular use. Advanced 3D models and developmental toxicology screening techniques might provide an answer to the need of nanotoxicological data, boosting the chances of translation of nanoparticles for ocular use.

In a large collaborative effort, Boada et al. developed a novel nanoparticle using low density lipoproteins (LDL) from plasma as building materials. The nanocarrier was named “aposome” due to the prevalence of apolipoprotein B (apoB) on its surface and was thoroughly characterized both physically and biologically. Following blood fractionation to obtain LDL, aposomes were prepared by thin layer evaporation/extrusion or microfluidic mixing of fractionated plasma with lipids. Microfluidic mixing enabled the development of smaller nanoparticles with a higher amount of incorporated apoB, compared to the thin layer evaporation/extrusion method. Interestingly, aposomes were comparable to liposomes in terms of loading capacity of the immunomodulator drug rapamycin while exhibiting a prolonged release profile. The authors assessed the biodistribution of aposomes in a genetic mouse model of atherosclerosis. Aposomes exhibited a 19-fold increase in plaque accumulation compared to untargeted liposomes, showing an increase of over two-fold compared to other previously published biomimetic nanosystems. The safety of aposomes was also scrutinized *in vitro* and *in vivo*. In general, aposomes administration did not affect liver or kidney functionality, did not induce the generation of acute phase or adaptive response antibodies, and did not increase cholesterol levels. Overall, aposomes represent a safe delivery nanoplatform that effectively overcomes the nonspecific distribution of nanoparticles by exploiting a biomimetic-based targeting approach that promotes the accumulation in specific pathological districts.

Each of these articles suggest possible approaches to overcome existing limitations of nanomedicines. There is consensus among the authors that attention should be devoted to assessing the biodistribution and safety of newly developed nanoparticles, in addition to exploiting biorelevant tools for their *in vitro* characterization. We hope that this Research Topic stimulates the interest of researchers and promotes a critical assessment of nanomedicines to harness their full potential as therapeutics and diagnostics.

